# The Role of Natural Killer Cells in the Tumor Immune Microenvironment of EBV-Associated Nasopharyngeal Carcinoma

**DOI:** 10.3390/cancers16071312

**Published:** 2024-03-28

**Authors:** Shuzhan Li, Wei Dai, Ngar-Woon Kam, Jiali Zhang, Victor H. F. Lee, Xiubao Ren, Dora Lai-Wan Kwong

**Affiliations:** 1 Department of Biotherapy, Tianjin Medical University Cancer Institute and Hospital, National Clinical Research Center for Cancer, Tianjin 300060, China; lishuzhan@tjmuch.com (S.L.); zhangjiali@tjmuch.com (J.Z.); 2 Tianjin’s Clinical Research Center for Cancer, Tianjin 300060, China; 3 Key Laboratory of Cancer Immunology and Biotherapy, Tianjin 300060, China; 4 Department of Clinical Oncology, Centre of Cancer Medicine, School of Clinical Medicine, LKS Faculty of Medicine, The University of Hong Kong, Hong Kong 999077, China; weidai2@hku.hk (W.D.); yvonnekam@hksccb.hk (N.-W.K.); vhflee@hku.hk (V.H.F.L.); 5 Laboratory for Synthetic Chemistry and Chemical Biology Limited, Hong Kong Science Park, New Territories, Hong Kong 999077, China; 6 Clinical Oncology Center, The University of Hong Kong-Shenzhen Hospital, Shenzhen 518053, China

**Keywords:** nasopharyngeal carcinoma, Epstein–Barr virus, natural killer cells, tumor immune microenvironment, anti-cancer therapy

## Abstract

**Simple Summary:**

Nasopharyngeal carcinoma (NPC) is an epithelial cancer associated with Epstein–Barr virus (EBV) infection. EBV infection contributes to not only the tumorigenesis of NPC but also the formation of a complicated tumor immune microenvironment (TIME) in NPC. Natural killer (NK) cells are lymphoid members of the innate immune system exhibiting both anti-tumor and anti-virus abilities. The cytokine and cellular interaction features of NK cells are distinct from those of CD8^+^ cytotoxic T cells. This unique characteristic has not been fully described in EBV^+^ NPC. This review provides an overview of the complicated crosstalk between NK cells and the TIME of EBV^+^ NPC and potential therapeutic strategies. A new perspective is proposed to direct future exploration.

**Abstract:**

Endemic nasopharyngeal carcinoma (NPC) is closely associated with the Epstein–Barr virus (EBV), which contributes to tumor development and influences the tumor immune microenvironment (TIME) in NPC. Natural killer (NK) cells, as part of the innate immune system, play a crucial role in responding to viral infections and malignant cell transformations. Notably, NK cells possess a unique ability to target tumor cells independent of major histocompatibility complex class I (MHC I) expression. This means that MHC I-deficient tumor cells, which can escape from effective T cell attack, are susceptible to NK-cell-mediated killing. The activation of NK cells is determined by the signals generated through inhibitory and activating receptors expressed on their surface. Understanding the role of NK cells in the complex TIME of EBV^+^ NPC is of utmost importance. In this review, we provide a comprehensive summary of the current understanding of NK cells in NPC, focusing on their subpopulations, interactions, and cytotoxicity within the TIME. Moreover, we discuss the potential translational therapeutic applications of NK cells in NPC. This review aims to enhance our knowledge of the role of NK cells in NPC and provide valuable insights for future investigations.

## 1. Introduction

**Figure 1:**
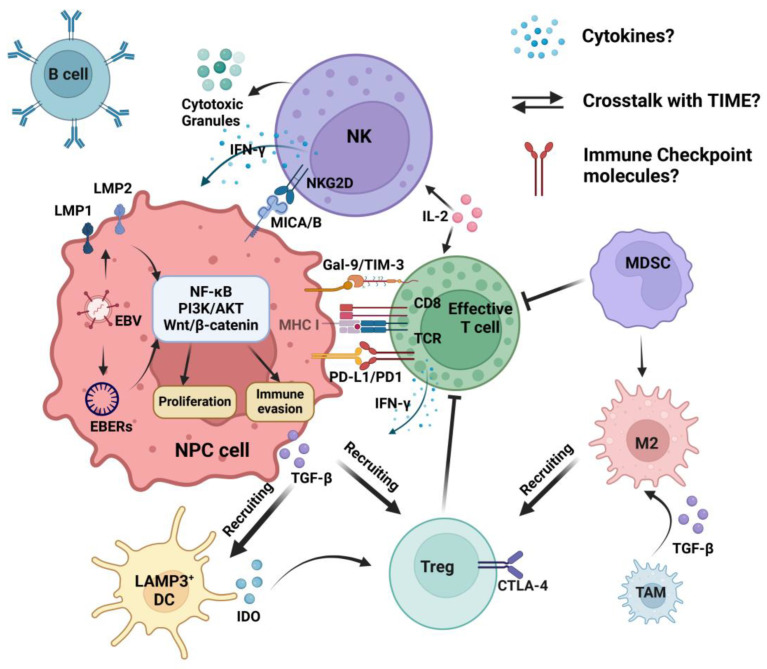
Tumor immune microenvironment of EBV^+^ NPC. EBV-associated LMP1/2 and EBERs activate NF-κB, PI3K/AKT, and Wnt/β-catenin pathways to induce the proliferation and immune evasion of NPC cells. TGF-β released by EBV^+^ NPC recruits immune-suppressive Tregs and LAMP3^+^ DCs. LAMP3^+^ DCs release IDO to promote Treg proliferation. TGF-β induces the M2 polarization of TAMs. MDSC promotes M2 proliferation. The effective T cell is inhibited by Treg and MDSC. T cell cytotoxicity is negatively regulated by the binding immune checkpoint receptors and their ligands. The NPC cell escapes the CD8^+^ T cell’s killing effect by diminishing MHC I expression. IL-2 activates the CD8^+^ T cell and NK cell, which are the major immune effectors. The B cell is a component within the TIME of EBV^+^ NPC. How NK cells interact with cytokines and other components and whether immune checkpoint molecules impact NK cell function need further discussion. Abbreviations: TIME, tumor immune microenvironment; EBV, Epstein–Barr virus; NPC, nasopharyngeal carcinoma; LMP1/2, latent membrane protein 1/2; EBERs, EBV-encoded small RNAs; TGF-β, transforming growth factor-β; Treg, regulatory T cell; LAMP3, lysosome-associated membrane glycoprotein 3; DC, dendritic cell; IDO, indoleamine-pyrrole 2,3-dioxygenase; TAM, tumor-associated macrophage; M2, M2-polarized TAM; MDSC, myeloid-derived suppressor cell; MHC I, major histocompatibility complex class I; IL-2, interleukin-2; NK, natural killer; NKG2, members of C-type lectin-like receptor superfamily, receptors of NK cells; MICA/B, major histocompatibility complex class-I-related chain A/B; IFN-γ, interferon-γ; Gal-9, galectin-9; TIM-3, T cell immunoglobulin domain and mucin domain-3; PD-1, programmed death-1; PD-L1, programmed death ligand-1; CTLA-4, cytotoxic T-lymphocyte antigen 4.

Nasopharyngeal carcinoma (NPC) is caused by both environmental and genetic factors [1]. According to GLOBOCAN estimates, there were 133,354 newly diagnosed patients and 80,008 mortalities due to NPC in 2020 [2]. NPC is more common in Southern China and Southeast Asia [3]. The non-keratinizing and undifferentiated histologic type accounts for 95% of NPC cases in the endemic area [4]. This subtype is highly associated with a childhood Epstein–Barr virus (EBV) infection. Other susceptibility factors include Cantonese ethnicity, smoking, diet habits, and several human leukocyte antigen (HLA) class I alleles [5]. Chemoradiotherapy is the standard first-line treatment for NPC, but approximately 20–30% of patients experience recurrence and/or metastasis (R/M) [6]. Immune checkpoint inhibitors (ICIs)—for instance, anti-programmed death-1 (PD-1) antibodies—have been proven to be safe and effective for R/M NPC patients [7]. This highlights the events happening in the tumor immune microenvironment (TIME) that will impact the patients’ outcomes. Immune evasion induced by cancer cells is currently considered a hallmark of cancer [8]. The TIME is a complicated environment due to the presence of various types of cells, including immunostimulant cells like CD8^+^ cytotoxic T cells (CTL) and natural killer (NK) cells; immunosuppressive cells, like regulatory T cells (Tregs); myeloid-derived suppressor cells (MDSCs); pro-inflammatory cytokines such as interferon-γ (IFN-γ); interleukin-2 (IL-2); and anti-inflammatory cytokines like transforming growth factor-β (TGF-β) and indoleamine-pyrrole 2,3-dioxygenase (IDO) (Figure 1) [9]. The TIME in EBV-associated NPC (EBV^+^ NPC) has distinctive features since latent EBV infection participates in the premalignant stage and promotes tumor progression [10].

The downregulation of HLA class I, which is required for the priming and activation of CTLs, is a common tumor evasion mechanism observed in EBV NPC [11,12]. EBV has been shown to suppresses major histocompatibility complex class I (MHC I) expression on NPC tumor cells, impairing the antigen presentation toward CD8^+^ T cells and promoting immune escape [13]. However, the loss of MHC I expression in NPC serves as a “missing self” signal that can be recognized by NK cells [14]. This provides an opportunity for NK cells to eliminate cancer cells since NK-cell-mediated cytotoxicity is not HLA-dependent and therefore may confer great clinical benefit to NPC treatment. As a critical part of immune surveillance, NK cells are lymphoid non-T cells that can kill virally infected and tumor cells [15,16]. Furthermore, NK cells bridge the innate and adaptive immune responses [17,18].

Therefore, understanding the crosstalk between NK cells and the TIME in EBV^+^ NPC is needed. In the present review, we summarized the vital role of NK cells in EBV^+^ NPC, reviewing multiple lines of in vivo and in vitro evidence indicating that NK cells and their subpopulation significantly contribute to the anti-EBV immune defense during NPC tumorigenesis as well as the anticancer potential of these cells.

## 2. EBV-Associated NPC and NK Cells

### 2.1. EBV-Associated NPC

EBV was the first identified oncogenic virus with a permanently infectious capacity in humans [19]. It is a double-stranded DNA virus, also one of the members of the human herpesvirus family [20]. EBV infection is associated with various epithelial and lymphoid malignancies, represented by NPC and Burkitt lymphoma, respectively [21,22]. EBV infects both B cells and epithelial cells [21,23]. It has been shown that NPC cells develop from a clonal proliferation of a single EBV-infected epithelial cell [24]. The tumorigenesis of EBV^+^ NPC may be mediated by EBV infection, carcinogens, and HLA genotype, collectively. EBV infection of nasopharyngeal epithelium is mediated by the interactions between ephrin receptor tyrosine kinase A2 (EphA2) and viral proteins gH/gL and gB [25,26]. The establishment and persistence of latency are crucial for the pathogenesis and tumorigenesis of EBV^+^ NPC [19]. Preexisting genome alterations support the switch from the lytic to latent phase [27,28]. This genome instability is considered a consequence of contact with carcinogens such as nitrosamines in salty fish, which are common in the Cantonese diet [10]. Genetic susceptibility factors include the HLA-A*0207 genotype, common among Chinese people, which is associated with NPC [29,30]. This HLA allele may not be capable of inducing a sufficient immune response against EBV viral antigens. With epithelial cells, only type II latency occurs [31]. This is confirmed by the expression of viral proteins restricted to type II latency, including EBV nuclear antigen 1(EBNA1), latent membrane protein 1 (LMP1), LMP2, and EBV-encoded small RNAs (EBERs) [32]. Latent proteins stimulate the NF-κB, PI3K/AKT, and Wnt/β-catenin signal pathways in NPC tumor cells (Figure 1). Downstream signal transduction promotes tumor cell proliferation and immune evasion [33,34,35,36,37]. Furthermore, LMP1 promotes an immune suppressive microenvironment by inducing the sensitivity of lymphocytes to TGF-β, which is well known as an immunosuppressive factor [38]. A small subset of NPC cells is detected with lytic EBV gene expression [13,39]. These minority NPC cells may induce immune evasion [40,41]. It has been proven that heavy immune cell infiltration and abundant secreted cytokines in EBV^+^ NPC establishes an immunosuppressive TIME [42], with a large population of Tregs, exhausted T cells, and MDSCs (Figure 1) [43,44].

### 2.2. NK Cells in NPC

NK cell expansion acts as the first responder in acute EBV infection, including in the acute phase of symptomatic EBV-associated infectious mononucleosis (IM) [45]. Several studies have indicated that NK cells not only contribute to the innate immune response against viral infection but also serve as a significant component in anti-cancer immune elimination [46,47]. High NK cell percentage in the TIME is associated with a better prognosis in EBV^+^ NPC [48,49]. Activated NK cells kill targeted cells by releasing perforin and granzyme and inducing antibody-dependent cell-mediated cytotoxicity (ADCC), and apoptosis [50,51]. NK cells’ function is modulated by the interactions between activating/inhibitory receptors and their ligands. The cytotoxic status will occur if there are more stimulatory signals aroused by activating receptors than inhibitory receptors, and vice versa [50]. Figure 1 illustrates the role of effective T cells in the TIME of EBV^+^ NPC. However, how NK cells interact with cytokines and other components within the TIME remains unclear. Whether immune checkpoint molecules impact NK cell function also needs further discussion.

## 3. NK Subpopulations in NPC

### 3.1. Surface Markers of NK Cells

**Figure 2:**
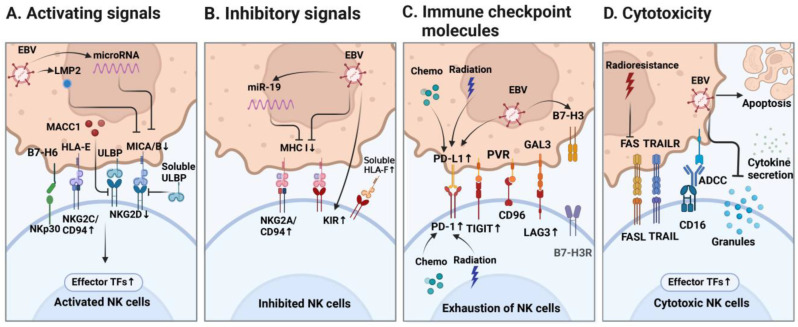
Profile of NK cells within the TIME of EBV^+^ NPC. (**A**) EBV^+^ NPC impairs NK cell activation; (**B**) EBV^+^ NPC promotes NK cell inhibition; (**C**) exhausted markers on NK cell; (**D**) cytotoxic function of NK cell is suboptimal. Abbreviations: EBV, Epstein–Barr virus; NPC, nasopharyngeal carcinoma; NK, natural killer; LMP2, latent membrane protein 2; miR, microRNA, a small non-coding RNA molecule; MACC1, metastasis-associated colon cancer 1; B7-H6, B7 homolog 6; HLA, human leukocyte antigen; ULBP, UL16 binding protein; MICA/B, major histocompatibility complex class-I-related chain A/B; CD94, killer cell lectin-like receptor subfamily D, member 1 (KLRD1), pairs with the NKG2 molecule as a heterodimer; NKp30, natural cytotoxicity triggering receptor 3; NKG2, members of C-type lectin-like receptor superfamily, receptors of NK cells; TFs, transcription factors; KIR, killer immunoglobulin-like receptor; MHC, major histocompatibility complex; TRAIL, TNF-related apoptosis-inducing ligand; TRAILR, receptor of TRAIL; FASL, Fas ligand; FAS, Fas receptor, apoptosis antigen 1; CD16, Fc receptors FcγRIII; ADCC, antibody-dependent cell-mediated cytotoxicity; PD-1, programmed death-1; PD-L1, programmed death ligand-1; TIGIT, T cell immunoreceptor with Ig and ITIM domains; PVR, polio virus receptor; CD96, T cell activation, increased late expression (TACTILE); LAG3, lymphocyte-activation gene 3; GAL3, galectin 3; B7-H3, B7 homolog 3, CD276; B7-H3R, receptor of B7-H3, not identified yet.

NK cells belong to innate lymphoid cells (ILCs) and exhibit anti-malignancy and anti-virus abilities. The typical biomarkers expressed on the NK cell surface are CD56^+^CD3^−^. The conventional categories of NK cells include CD56^dim^CD16^+^ (“mature” cytotoxic NK cells) and CD56^bright^CD16^−^ (“immature” immunomodulatory NK cells). CD56^dim^CD16^+^ NK cells are the predominant component, producing abundant IFN-γ, tumor necrosis factor-α (TNF-α), and cytolytic mediators perforin and granzymes. CD56^bright^CD16^−^ NK cells have poorer cytotoxic capability but can be activated within a pro-inflammatory microenvironment [52]. NKG2D and NKp30 are typical markers expressed on an activated NK cell’s surface, and the ligands are MHC Class-I-related Chain A (MICA) and MICB for the former and B7-H6 for the latter (Figure 2A) [53,54]. Meanwhile, NKG2A/CD94 heterodimers and killer immunoglobulin-like receptors (KIR) are inhibitory receptors that recognize the MHC I molecules expressed by host cells (Figure 2B). “Missing self recognition” will occur between activated NK cells and aberrant host cells with MHC I loss, then NK cells perform as innate immune defenders without antigen priming [55,56].

### 3.2. NK Cells in Peripheral Blood of NPC Patients

The percentage of NK cells out of all peripheral blood mononuclear cells ranges from 1% to 15% [57,58]. Regarding the subtypes of NK cells, it has been revealed that there is no difference between the percentage of NK cells in peripheral lymphocytes isolated from healthy individuals and NPC patients [59,60]. One study indicated that there was no difference between the percentage of NKG2D^+^ NK cells (activated) and KIR2DL2/DL3^+^NK2GA^+^ NK cells (inhibited) in peripheral blood samples of NPC patients and a healthy control. However, a lower NKp30^+^ NK cell percentage is noticed in NPC patients than in healthy controls [59]. A similar result has been discovered in another study, in which a lower NKG2D^+^ NK cell percentage was found when comparing NPC patients with healthy individuals [61]. These may indicate a poorer NK cell anti-cancerous function in NPC patients.

### 3.3. NK Cells in the TIME of EBV^+^ NPC

The distribution of NK cells in the TIME is a crucial it performing its role as an anti-cancer immune defender. In tumor tissue samples from an NPC biopsy, the NK cells account for 1–6.5% of all stromal cells regardless of their subtypes [12,44,48]. Chen and colleagues found that NK cells were enriched in the tumor stroma, with lower infiltration in the tumor epithelial nests [48]. Studies have also indicated that compared with nonmalignant nasopharyngeal biopsies, NK cells showed an increased trend in EBV^+^ NPC tissue [62,63]. Unfortunately, these studies have not indicated NK cell subtypes. On the contrary, when detected with immunohistochemical staining (IHC), CD56^+^ NK cells were reduced in non-keratinizing NPC tissue compared with normal nasopharyngeal mucosa [64]. Peng and colleagues demonstrated that CD56^dim^CD16^+^ NK cells, the cytotoxic subtype, accounted for the majority of NK cells in both treatment naïve and recurrent EBV^+^ NPC tissues. Significantly, CD56^dim^CD16^+^ NK cells exhibited more cytotoxicity and chemotaxis and a more exhausted status in recurrent than primary NPC samples [12]. This may suggest that the composition of NK cells can be modulated by both treatment methods and interactions between cancer cells and the immune system.

According to single-cell transcriptomic and proteomic analysis research, infiltrated NK cells within EBV^+^ NPC biopsies could be divided into three sub-groups, which showed cytotoxic, inhibited, and exhausted phenotypes, respectively. Notably, these exhausted NK cells showed an upregulation of both lymphocyte-activation gene 3 (LAG3) and T cell immunoreceptor with Ig and ITIM domain (TIGIT) expression, both known as immune checkpoint receptors [64]. Also, comparing the EBV^+^ NPC and EBV^−^ NPC biopsies, the percentage of activated CD56^+^CD3^−^ granzyme B^+^ NK cells was found to be lower for EBV^+^ NPC [65]. Overall, the subpopulations of NPC-infiltrated NK cells remain unclear. However, it seems that most NK cells within EBV^+^ NPC tissue express inhibitory or exhausted biomarkers, indicating a consequence of immune evasion.

## 4. The Crosstalk of NK Cells in the TIME

The functional status of NK cells is identified by the interaction between functional molecules expressed on the NK cell surface and their receptors/ligands. It can be divided into four types: where cell–cell interaction induces (1) an activating signal; (2) an inhibitory signal; (3) signals initiated by cytokines/chemokines; or (4) interaction turning on the apoptotic downstream signal or antibody-dependent cellular cytotoxicity (ADCC) in targeted cells [50]. Table 1 summarizes the relevant NK functional receptors and ligands that have been reported in NPC.

### 4.1. Activating and Inhibitory Signals of NK Cells

Tumor cells, other immune cells, and cytokines are capable of binding with the molecules expressed on NK cells, triggering activation or inhibition status. This complicated network needs further investigation. Figure 2 summarizes the activating and inhibitory signals affecting NK cells. Notably, EBV-derived latent protein LMP2 and microRNA suppress MICA/B expression, which are ligands binding to NKG2D (activating receptor) on NK cells [66,67,68]. Meanwhile, EBV performs as a “double-edged sword” in generating inhibitory signals affecting NK cells. EBV downregulates MHC I molecule expression to avoid effective CD8^+^ T cell attack but arouses “missing self” recognition by NK cells [13,76]. Persistent exposure to the EBV^+^ cell line promotes inhibitory KIR expression on NK cells ex vivo [77]. Therefore, the terminal NK functional status in the TIME of NPC may not be described precisely.

### 4.2. Cytokines/Chemokines and Immune Cells

**Figure 3:**
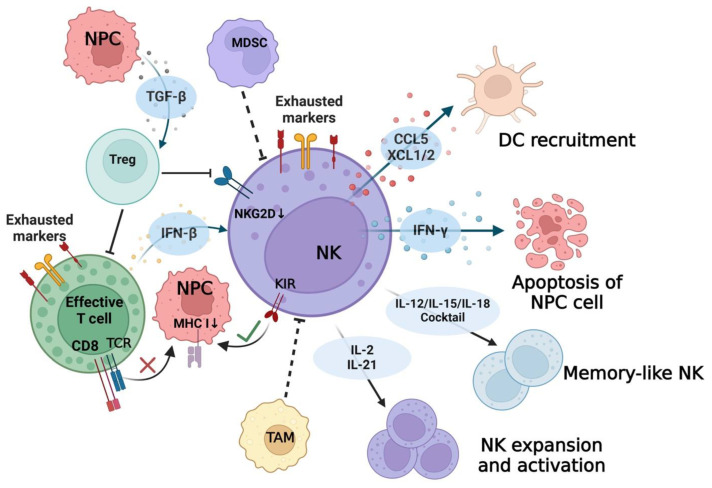
Interaction between NK cells and cytokines, chemokines, and other immune cells. NK-cell-derived CCL5 and XCL1/2 play a vital role in recruiting DCs; IFN-γ released by NK cells induces the apoptosis of NPC cells. IL-12, IL-15, and IL-18 cocktail cytokines convert NK cells into “memory-like” NK cells. IL-2 and IL-21 promote strong NK cell expansion and cytotoxicity. “×” The cytotoxicity of CD8^+^ T cells is inhibited through MHC I downregulation in EBV^+^ NPC. “√” The NK cell is activated via “missing self” recognition of downregulated MHC I expression on EBV^+^ NPC. Effective T cells release pro-inflammatory cytokine IFN-β to enhance NK cell cytotoxicity. Abbreviations: NPC, nasopharyngeal carcinoma; TGF-β, transforming growth factor-β; MDSC, myeloid-derived suppressor cell; Treg, regulatory T cell; NK, natural killer; IFN, interferon; TCR, T-cell receptor; MHC I, major histocompatibility complex class I; NKG2, members of C-type lectin-like receptor superfamily, receptors of NK cells; KIR, killer immunoglobulin-like receptor; CCL5, chemokine (C–C motif) ligand 5; XCL1/2, chemokine C motif ligand 1/2; DC, dendritic cell; TAM, tumor-associated macrophage.

Cytokines in the TIME show either stimulating or inhibitory patterns in the modulation of NK cell function (Figure 3). EBV^+^ NPC releases TGF-β, which inhibits NK cell function by promoting the differentiation of Tregs [77]. On the other hand, immune cells such as T cells express IFN-β in the TIME which promotes NK cell function via the TRAIL signaling pathway [83]. Chemokines like CCL5 and XCL1/2 are secreted by activated NK cells and recruit antigen-presenting dendritic cells (DCs) [18,44,48,93]. These molecules facilitate the interaction between NK cells and other components within the TIME. Tumor-associated macrophages (TAMs) and MDSCs have an inhibitory effect in the TIME of EBV^+^ NPC [91]. It has been reported that TAMs and MDSCs inhibit NK cell function to facilitate the immune escape of solid tumors [62,97]. The immune suppressive role of TAMs and MDSCs towards NK cells within EBV^+^ NPC needs further investigation.

It has been well defined that the pro-inflammatory cytokines IL-2 and IL-21 promote NK cell expansion [50,90,91]. The IL-12, IL-15, and IL-18 cytokine cocktail can convert the cells into long-lived, activated “memory-like NK cells” [90]. Based on this, therapeutic applications for NPC patients, such as adoptive NK cells combined with other immune stimulators, may trigger effective and steerable immune responses against NPC.

### 4.3. Immune Checkpoint Molecules

Notably, immune checkpoint receptors are involved in the modulation of NK cell function, of which the ligands expressed on NPC cells are also elevated to establish immune evasion (Figure 2C). Although EBV infection will induce high levels of the production of the pro-inflammatory cytokine IFN-γ by NK cells and cytotoxic T cells, persistent IFN-γ exposure in EBV^+^ NPC can promote the expression of exhausted biomarkers like PD-1 [62]. EBV increases PD-L1 expression in NPC cells via EBV-encoded miRNA [97]. Conventional chemotherapy and radiotherapy promote PD-L1 expression on NPC cells and PD-1 expression on NK cells [78,79]. Other novel immune checkpoint molecules like TIGIT, LAG3, and CD96 have been found expressed on exhausted NK cells in NPC [44,48,63]. The downstream effects of these exhausted markers on NK cells needs to be further investigated in EBV^+^ NPC.

## 5. NK Cell Cytotoxicity

NK cells are well known as a type of cytotoxic lymphocyte critical to the innate immune system. Normally, NK cell cytotoxicity involves several mechanisms including triggering apoptosis in targeted tumor cells, inducing ADCC, or the secretion of cytokines that recruit or stimulate other immune cell types (Figure 2D). Once NK cells are activated, the pore-forming protein perforin and the granzyme B contained in cytotoxic granules are released [98]. In the meantime, NK cells activate the death receptors FAS and TRAIL expressed on targeted tumor cells and induce apoptosis [99]. CD16 and CD32 are high-affinity Fc receptors expressed on the NK cell surface. Antibody-coated cells will be killed by NK cells once CD16/CD32 binds with the Fc region of the antibody [100]. IFN-γ and TNF-α are released by stimulated NK cells, which can induce tumor cell death [101]. Otherwise, NK cells participate in the stimulation of the adaptive immune system by recruiting other effective cells or activating antigen-presenting procedure (Figure 3) [17,18].

### 5.1. NK Cells against EBV Infection

Traditionally, the adaptive immune response is considered the primary defense against virus infections. However, it has been revealed that virus-specific T cells alone are not sufficient to eliminate EBV infection [102,103]. Patients with NK cell deficiency experience severe symptoms following EBV infection and are at a higher risk of developing EBV-related malignancies [103]. In summary, circulating CD56^dim^NKG2A^+^KIR^−^ NK cell expansion may act as the first responder in acute EBV infection, as in the acute phase of symptomatic EBV-associated IM [45]. Several studies have indicated that NKG2A^+^ NK cells are significant effector cells against EBV-infected B cells [45,104]. Notably, it has been found that the latent phase of EBV infection is more resistant to NK-cell-mediated cytotoxicity compared to the lytic phase. This could be due to the downregulation of MHC I and upregulation of NK-activated ligands that occur during lytic EBV infection in B cells [105]. MHC I expression is regained after switching to the latent phase [45]. Additionally, the expression of lytic-phase genes carried by EBV makes EBV-infected B cells more susceptible to NK-cell-mediated killing [105,106]. The lifelong carrying of latent EBV infection in resting B cells may indicate a balance between the host cells and NK-cell-mediated killing processes.

### 5.2. NK Cells against EBV^+^ NPC

Since type II latent phase EBV infection is predominant in EBV^+^ NPC, it is more likely that the cytotoxic function of NK cells remains quiescent in the TIME. A stronger NK cell expansion and cytotoxicity could be induced in NPC-patient-derived NK cells in comparison with healthy controls and 5-year NPC survivors [60]. This could be explained by the higher production of pro-inflammatory cytokines (IL-12, IL-15, and TNF-α) but not the effector cytokine (IFN-γ) by cultured NPC-patient-derived NK cells ex vivo [60]. The activating signal for NK cells may not be sufficient to arouse an anti-tumor response. Gong and colleagues found that NK cells expressed a high level of cytotoxic genes in NPC [44]. However, a proteomic analysis revealed that the function of NK cells in NPC tissues was inhibited. This was confirmed by decreased CD56 and granzyme B IHC staining. Further investigation showed that NK cell downregulation is due to exhaustion induced by NPC tumor cells [64]. EBV infection may contribute to this downregulation of NK cell function. Decreased NK cell degranulation was exhibited in cocultured NK cells with EBV^+^ NPC cells when compared with their parental group, but not when cocultured with EBV^−^ NPC cells ex vivo [65]. This indicated that NK cell cytolytic activity was inhibited by EBV^+^ NPC cells directly. This may be partially due to the upregulation of B7-H3, an immune checkpoint molecule that is also a member of B7 superfamily, mediated by EBV infection [65]. A lower expression of immune signatures in NK cells was found to be associated with EBV^+^ NPC when compared with EBV^−^ NPC samples. Interestingly, in the same study, patients with a higher expression of immune signatures in NK cells had a better PFS [48]. Transcription factors (TFs) such as XBP1, EOMES, and RUNX3 were upregulated in NK cells. The same upregulated expression patterns were detected in the cytotoxic CD8^+^ T cell subtypes. To validate this result, NK cells and CD8^+^ T cells were isolated from NPC tissue samples. A higher percentage of cytotoxic granzyme B^+^ or perforin^+^ phenotype was verified in the XBP1^+^, EOMES^+^, and RUNX3^+^ NK cells and CD8^+^ T cells [48]. This study illustrated the significant effective role of NK cells and CD8^+^ T cells in the TIME for anti-tumor immune response. The “missing self” recognition of NK cells acts as a functional supplement to overcome the CD8^+^ T cell off-target tumor killing mediated by MHC I downregulation in EBV^+^ NPC. Meanwhile, activated immune cells secrete pro-inflammatory cytokines like IFN-β to enhance NK cell cytotoxicity (Figure 3).

To sum up, these inconsistent results suggests that NK cells within the NPC tumor site ensure an inhibitory, exhausted, and suboptimal status, more like a precursor for activated and cytotoxic NK cells. Overcoming this phenomenon could be used as a strategy to improve NPC-directed therapeutic methods.

## 6. NK Cells in Cancer Therapy

It is important to understand the initiating, expansion, recruiting, and activation/inhibition process, and the functional status of NK cells in NPC to further investigate treatment strategies. NK cells establish a relatively lower, more inhibitory, and exhausted subpopulation in the TIME of NPC. To overcome this, adoptive NK cell therapy with ex vivo expansion and pre-stimulation or with genetically engineering can be considered. Therapies that activate NK cells in vivo alone or combined with adoptive NK cells may also be effective. One advantage of adoptive NK cell therapy is that activated NK cells are not HLA-matching-dependent and do not mediate graft-versus-host disease (GVHD) [107]. Based on this, NK cell products can be used in allogeneic recipients, or designed to be commercially available.

### 6.1. Pre-Activated/Genetically Modified Adoptive NK Cells

In early investigations, the adoptive transfer of partially allogenic NK cells isolated from peripheral blood and co-cultured with IL-2 was proven to have anti-cancer capability with low toxicity in patients with acute myelocytic leukemia [108]. NK cells isolated from peripheral blood were pre-stimulated ex vivo with an IL-12, IL-15, and IL-18 cytokine cocktail to induce “memory-like” NK cells [109]. These “memory-like” NK cells exhibited higher IFN-γ production and expression of granzyme B and perforin. Several clinical trials based on these “memory-like” NK cells are ongoing [110,111,112]. However, these trials are focusing on hematologic tumors. Solid tumors like NPC treated by pre-stimulating NK cells with cytokines have limited efficacy. One approach to enhance the effective function of NK cells is to use NK cells armed with monoclonal antibodies to target molecules expressing on the surface of tumor cells, so-called “armed NK cells” [113]. It is reported that EGFR is highly expressed in most NPC and is a poor prognostic factor [114]. Cetuximab, a monoclonal antibody against EGFR, was added to the cultural system for autologous NK cells from NPC patients. CD16 expressed on NK cells recognized the Fc region of Cetuximab. This induced ADCC in NK cells against C666 NPC cells ex vivo [115]. Concurrent treatment of cetuximab plus autologous NK cells in EGFR-positive NPC was tolerable among R/M NPC patients. Durable stable disease was observed in three out of seven subjects (NCT02507154) [116]. In addition, the limited anti-cancer function of NK cells in solid tumors may partially be due to poor trafficking toward tumor nests. The genetically engineered transduction of chimeric antigen receptor (CAR) to NK cells increases the capability of recognition of NK cells to targeted tumor antigens. CD137, a costimulatory receptor expressed on T cells, was found to exhibit ectopic expression in 42 of 122 (34.4%) NPC cases, induced by EBV-related LMP1 expression. A negative feedback mechanism hijacked by NPC cells downregulated the expression of the ligand of CD137 (CD137L), causing immune evasion [117]. NK cells express CD137-specific CAR-induced death in CD137^+^ NPC cells, in vitro. CD137-specific CAR NK cells established an anti-tumor effect in a murine xenograft model [117]. Further investigation for safety and efficacy in CD137^+^ NPC patients is needed.

### 6.2. Adoptive NK Cells Combined with Systemic/Conventional Therapies

Evidence is cumulating to confirm that NK cells upregulate PD-1 in certain cancers [118,119]. PD-1 blockades cause an anti-tumor NK cell response, which is essential to ensure the therapeutic effect of anti-PD-1 therapy [120]. The inhibition of PD-1 was proven to increase cytotoxicity towards NPC cells by activated NK cells [78]. The combination of IFN-β and anti-PD-1 augmented the cytolytic effect of NK cells against NPC cells [94]. TIGIT has been found to be overexpressed in the exhausted NK cell subset in the TIME of NPC [64]. The blockade of TIGIT with monoclonal antibodies reverted NK cell exhaustion to functional status in multiple tumor models [121]. A combination of PD-1 or TIGIT blockade with adoptive NK cell therapy may have an optimal therapeutic effect in NPC patients. These results need further investigation in vivo and in clinical trials.

Chemotherapies such as gemcitabine, cisplatin, and 5-fluorouracil can sensitize NPC cells to NK cell cytotoxicity. These chemotherapeutic agents also induce the expression of programmed death ligand-1 (PD-L1) on NPC cells [78]. Meanwhile, radiotherapy sensitizes NPC cells to NK cell killing. Radiation can induce PD-L1 expression in NPC cells and PD-1 expression in NK cells [79]. Conventional therapies such as chemotherapy and radiotherapy combined with ICIs and with/without adoptive NK cells may be a therapeutic choice for NPC patients after failing of systemic therapy.

### 6.3. NK Cell Agonists

NK cell activity can be strongly expanded by innate immune activators, such as the synthetic double-stranded RNA analog poly(I:C), a Toll-like receptor 3 (TLR3) agonist [122]. Poly(I:C) enhanced ADCC effect on NPC cells after overnight treatment of immature NK cells from NPC patients [115]. Additionally, poly(I:C)-treated NK cells established stronger degranulation and IFN-γ secretion after they were co-cultured with C666 and cetuximab [116]. The treatment of peripheral blood mononuclear cells (PBMC) with a selective TLR8 agonist VTX-2337 in vitro activated NK cells, enhanced cetuximab-mediated ADCC, and augmented tumor killing [123,124]. A phase I dose escalation open-label trial enrolled 13 patients with recurrent or metastatic squamous cell carcinoma of the head and neck (SCCHN), including NPC. The patients’ NK cells became more responsive to NKG2D stimulation following VTX-2337 treatment (NCT01334177) [125].

In addition, cytosolic DNA generated from viral DNA and chromosomal instability of cancer is thought to trigger the cyclic GMP-AMP synthase (cGAS) stimulator of interferon genes (STING) pathway [126]. The activation of the cGAS-STING pathway supports the initiation of NK cell response to viral infection and tumors [127]. Therefore, STING agonists mobilize powerful NK-cell-mediated antitumor response [128]. TAK-500, a novel STING agonist, is currently being evaluated as a single agent and in combination with pembrolizumab for tolerability and preliminary antitumor activity in adult patients with select locally advanced or metastatic solid tumors, including NPC (NCT05070247).

As described earlier in this review, NK cells express inhibitory receptors KIR and NKG2A. It has been reported that KIR and NKG2A are inhibitory receptors expressed by NK cells in EBV^+^ NPC [12]. Blocking KIR and NKG2A may have therapeutic potential in EBV^+^ NPC. Monoclonal antibodies against KIR and NKG2A have unleashed NK cell function in preclinical studies and multiple tumor models [129,130]. Anti-KIR antibodies and anti-NKG2A antibodies as monotherapy or combined with ICIs may arouse the persistent activation of NK cells [129,131]. The efficacy and safety need further investigation in clinical trials.

### 6.4. NK Cell Engager

Nowadays, an alternative approach, the NK cell engager, is being investigated to amplify NK cell function against tumors. The engager bridges NK-cell-activating receptors and molecules expressed on tumor cells. One arm of the engager binds the activating NKp46 receptor and the activating Fc receptor CD16 on NK cells. The other arm binds to tumor-associated antigens, including CD19, CD20 for hematologic tumors, and EGFR for solid tumors [50,132]. A bispecific engager with high affinity and specificity toward CD16 and human epidermal growth factor receptor 2 (HER2) was designed and tested. A higher ADCC effect was induced in HER2^+^ breast cancer cells in comparison to anti-HER2 monoclonal antibody trastuzumab [133]. This novel approach needs to be further tested in vivo and in clinical trials. Meanwhile, an optimal tumor antigen presented by NPC cells also needs to be determined for NK cell engager design.

The immunosuppressive and exhausted condition of NK cells in the TIME needs to be reversed to fully develop anti-cancer effectiveness. Table 2 summarizes the registered clinical trials relevant to NK cells in NPC treatment. Multiple investigations are necessary for the translational applications of NK cells in NPC in the future.

## 7. Conclusions and Future Directions

There has been impressive progress in the understanding of the TIME in EBV^+^ NPC. NK cells, as members of innate immune defenders, have been investigated deeply in recent years. In this review, we summarized the subpopulations, crosstalk with cytokines/chemokines and other components, and the functional status of NK cells in the TIME of EBV^+^ NPC. NK cell response is initiated after EBV infection. Then NK cell population expands and expresses several activating biomarkers like NKG2D and CD16 and inhibitory biomarkers like NKG2A/CD94 and KIR. Whether the cytotoxicity of NK cells is activated or inhibited is determined by the stronger signal generated by activated or inhibitory receptors. This procedure occurs in an early stage of EBV infection, even before premalignancy transformation. Finally, when the tumor is developed, NK cells present more insufficient and exhausted conditions in the TIME. Therapeutic methods such as exogenous NK cell infusion and agents stimulating endogenous NK cell function can be designed for further investigation to fully mobilize the anti-cancer function of the enriched immune cells infiltrated in the TIME of EBV^+^ NPC.

## Figures and Tables

**Table 1:** Receptors and ligands relevant to NK cells in NPC.

Molecules on NK Cells	Corresponding Receptors/Ligands	Functional Signal Reported in NPC	References
(1) Activating signaling			
NKG2D	MICA/B ULBP	LMP2 induced MICA/B downregulation in NPC.	[66]
		EBV-encoded mircoRNAs promoted MICA/B downregulation in NPC.	[67,68]
		MICA gene deletion was found in NPC.	[69,70]
		Free soluble ULBP protein secreted by NPC cells impaired NK function.	[71]
		Over-expression of MACC1 on NPC cells reduced NKG2D expression on NK cells.	[61]
NKp30	B7-H6	A low percentage of NKp30^+^NK cells was found in NPC tissues.	[59]
NKG_2C_/CD94	HLA-E	CD94 expression on NK cells was increased in NPC; NKG2C-HLA-E signaling pathway was not relevant to genetic susceptibility in NPC.	[44,72]
(2) Inhibitory signaling			
KIR	MHC I	HLA-C (tumor cell) and KIR2DL4 (NK cell) interaction was reported in NPC.	[62,73]
		Higher HLA-G expression was found in NPC when compared with normal tissue.	[74]
		Higher soluble HLA-F was found in NPC plasma than normal controls.	[75]
		miR-19 reduced MHC I molecule expression on NPC cell line.	[76]
		Persistent exposure to CNE2 cell line increased KIR expression on NK.	[77]
NKG2A/CD94		NKG2A expression of NK cells was not upregulated in NPC; HLA-E (tumor cell) and CD94 (NK cell) interaction was found in NPC.	[44,62]
PD-1	PD-L1	B7-H3 knockdown in EBV^+^ NPC cell line increased PD-1 expression on cocultured NK cells.	[65]
		Chemotherapy upregulated PD-1 on NK cells and PD-L1 on NPC cells via NF-κB pathway.	[78]
		Radiotherapy for NPC increased PD-1 expression on NK cells and PD-L1 expression on tumor cells.	[79]
		PD-1 expression on NK cells in NPC could be induced by IL-18.	[80]
		Soluble PD-L1 was detected in NPC plasma, indicating poor prognosis.	[81]
TIGIT	PVR	TIGIT-PVR interaction was detected between NK and tumor cells and between NK and macrophages/dendritic cells in NPC; TIGIT expression was upregulated on exhausted NK cells in NPC.	[48,64]
		CD96-PVR interaction was detected between NK and tumor cells in NPC	
CD96		TIGIT and CD96 were positively correlated with FCER2 and KHDRBS2 and negatively correlated with IGSF9 in NPC *.	[63]
LAG3	Gal-3	LAG3 expression was upregulated on exhausted NK cells in NPC.	[64]
		LAG3 was positively correlated with FCER2 and KHDRBS2 and negatively correlated with IGSF9 in NPC *.	[63]
(3) NK-cell-related cytokines/chemokines			
TGF-β		TGF-β inhibited NK cell function by promoting Tregs differentiation; TGF-β promoted the transmission of EBV infection in NPC.	[82]
Type I Interferon		Type I Interferon induced granzyme B directly in NK cells; IFN-β induced NK-cell-mediated cytotoxicity against NPC targets in vitro.	[83,84]
IL-2		IL-2 promoted strong NK cytotoxicity.	[50]
IL-12		IL-12 and IL-18 stimulated IFN-γ production by NK cells synergistically; IL-12 secretion was related to EBV viral stimuli; the anti-EBV NK subset could be activated by IL-12.	[85,86,87]
IL-15		IL-15 promoted strong NK cytotoxicity and ADCC and induced the expression of NKp30.	[88,89]
IL-18		A negative correlation between IL-18 and NK cytotoxicity in NPC was reported; IL-18 level increased during EBV infection. IL-12, IL-15, and IL-18 cocktail cytokines converted NK cells into long-lived, activated NK cells called “memory-like” NK cells.	[80,90]
IL-21		IL-21 promoted the expansion of NK cells in vitro; IL-21 expression elevated on follicular TLSs in NPC.	[91,92]
XCL1/2		Interaction between XCR1 and XCL1/2 was involved in DCs recruited by NK cells; XCL1/2 were overexpressed by NK cells in NPC.	[18,44,48]
CCL5		NK-cell-derived CCL5 played a vital role in recruiting DCs; CCL5 was identified as an EBV-regulated molecule driver promoting NPC angiogenesis.	[93]
(4) Functional signal inducing apoptosis in target cells			
TRAIL	TRAILR	Binding of TRAIL and TRAILR activated downstream death signal; TRAIL sensitivity was redox-dependent in NPC cells; NK-cell-dependent NPC cell killing was predominately mediated via TRAIL pathway.	[94]
FASL	FAS	FAS/FASL signal triggered apoptosis in target cells; FAS expression was suppressed in radioresistant NPC patients.	[95]
CD16	Fc region	CD16 was considered as an activated biomarker for NK cells; CD16/CD32 binding with Fc region of IgG antibody triggered ADCC effect; ADCC participated in anti-EBV infection in lytic phase.	[96]

* FCER2, KHDRBS2, and IGSF9 were found to be associated with NPC [63]. Abbreviations: NK, natural killer; NPC, nasopharyngeal carcinoma; NKG2, members of C-type lectin-like receptor superfamily, receptors of NK cells; MICA/B, major histocompatibility complex class-I-related chain A/B; ULBP, UL16 binding protein; LMP2, latent membrane protein 2; EBV, Epstein–Barr virus; MACC1, metastasis-associated colon cancer 1; NKp30, natural cytotoxicity triggering receptor 3, CD337; B7-H6, B7 homolog 6; HLA, human leukocyte antigen; KIR, killer immunoglobulin-like receptor; MHC, major histocompatibility complex; miR, microRNA, a small non-coding RNA molecule; CD94, killer cell lectin-like receptor subfamily D, member 1 (KLRD1), pairs with the NKG2 molecule as a heterodimer; PD-1, programmed death-1; PD-L1, programmed death ligand-1; B7-H3, B7 homolog 3, CD276; IL, interleukin; TIGIT, T cell immunoreceptor with Ig and ITIM domains; PVR, polio virus receptor; CD96, T cell activation, increased late expression (TACTILE); LAG3, lymphocyte-activation gene 3; Gal-3, galectin 3; TGF-β, transforming growth factor-β; IFN-β, interferon-β; TLSs, tertiary lymphoid structures; DCs, dendritic cells; XCL1/2, chemokine C motif ligand 1/2; XCR1, the receptor for XCL1/2; CCL5, chemokine (C–C motif) ligand 5; TRAIL, TNF-related apoptosis-inducing ligand; TRAILR, receptor of TRAIL; FASL, Fas ligand; FAS, Fas receptor, apoptosis antigen 1; CD16, Fc receptors FcγRIII; CD32, Fc receptors FcγRII.

**Table 2:** Registered clinical trials associated with NK cells.

Phase	Treatment	Condition	Estimated Enrollment	Completion Date	Status	NCT No.
Phase I	IL-2 and NK cells	Metastatic NPC	Not mentioned	Not mentioned	Completed	NCT00717184
Phase I/II	Cetuximab with NK cells	NPC	31	1 August 2019	Unknown	NCT02507154 [116]
Phase I/II	High-activity natural killer immunotherapies	Small metastatic NPC	20	1 June 2019	Completed	NCT03007836
Phase I	Haplo/Allogeneic NKG2DL-targeting CAR-grafted γδ T Cells	Relapsed or refractory solid tumors including NPC	10	1 March 2021	Unknown	NCT04107142
Phase I	VTX-2337 with cetuximab	Locally advanced, recurrent, or metastatic SCCHN including NPC	13	Not mentioned	Completed	NCT01334177 [125]
Phase I/II	TAK-500 with or without pembrolizumab	Locally advanced or metastatic solid tumors including NPC	313	11 August 2026	Recruiting	NCT05070247

Abbreviations: NK, natural killer; NPC, nasopharyngeal carcinoma; IL, interleukin; NKG2, members of C-type lectin-like receptor superfamily, receptors of NK cells; CAR, chimeric antigen receptor; SCCHN, squamous cell carcinoma of the head and neck.

## Data Availability

No new data were created or analyzed in this study. Data sharing is not applicable to this article.
